# Phenyl pyrazin-2-yl ether

**DOI:** 10.1107/S1600536808040099

**Published:** 2008-12-03

**Authors:** Azila Idris, Azhar Afiffin, Zanariah Abdullah, Seik Weng Ng

**Affiliations:** a Department of Chemistry, University of Malaya, 50603 Kuala Lumpur, Malaysia

## Abstract

In the title compound, C_10_H_8_N_2_O, the dihedral angle between the aromatic rings is 64.2 (1)° and the bridging C—O—C angle is 119.1 (1)°.

## Related literature

For the structure of quinoxalinyl phenyl ether, see: Hassan *et al.* (2008 ▶). For the structure of *N*-(pyrazin-2-yl)aniline, see: Wan Saffiee *et al.* (2008 ▶).
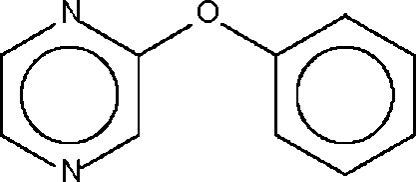


## Experimental

### 

#### Crystal data

C_10_H_8_N_2_O
*M* *_r_* = 172.18
Monoclinic, 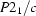

*a* = 5.704 (1) Å
*b* = 8.557 (2) Å
*c* = 17.595 (4) Å
β = 94.382 (3)°
*V* = 856.4 (3) Å^3^
*Z* = 4
Mo *K*α radiation
μ = 0.09 mm^−1^
*T* = 100 (2) K
0.20 × 0.15 × 0.10 mm

#### Data collection

Bruker SMART APEX diffractometer
Absorption correction: none
4641 measured reflections
1950 independent reflections
1207 reflections with *I* > 2σ(*I*)
*R*_int_ = 0.042

#### Refinement

*R*[*F* ^2^ > 2σ(*F* ^2^)] = 0.046
*wR*(*F* ^2^) = 0.119
*S* = 0.97
1950 reflections
118 parameters
H-atom parameters constrained
Δρ_max_ = 0.20 e Å^−3^
Δρ_min_ = −0.27 e Å^−3^

### 

Data collection: *APEX2* (Bruker, 2007 ▶); cell refinement: *SAINT* (Bruker, 2007 ▶); data reduction: *SAINT*; program(s) used to solve structure: *SHELXS97* (Sheldrick, 2008 ▶); program(s) used to refine structure: *SHELXL97* (Sheldrick, 2008 ▶); molecular graphics: *X-SEED* (Barbour, 2001 ▶); software used to prepare material for publication: *publCIF* (Westrip, 2008 ▶).

## Supplementary Material

Crystal structure: contains datablocks global, I. DOI: 10.1107/S1600536808040099/hb2867sup1.cif

Structure factors: contains datablocks I. DOI: 10.1107/S1600536808040099/hb2867Isup2.hkl

Additional supplementary materials:  crystallographic information; 3D view; checkCIF report

## Figures and Tables

**Fig. 1.**
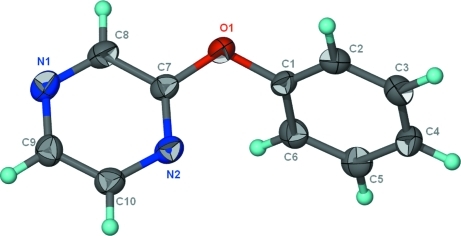
The molecular structure of (I) at the 70% probability level; hydrogen atoms are drawn as spheres of arbitrary radius.

## supplementary crystallographic information

### Experimental

Phenol (0.94 g, 0.01 mol) was dissolved in a small volume of water containing sodium hydroxide (0.40 g, 0.01 mol). The mixture was heated to remove most of the water. This and 2-chloropyrazine (1.15 g, 0.01 mol) were heated for 5 h. The material was extracted with chloroform and the organic phase then dried over sodium sulfate. Evaporation of the solvent gave the crude product, which was recrystallized from chloroform.

### Refinement

The H atoms were placed in calculated positions (C—H = 0.95 Å) and refined as riding with *U*_iso_(H) = 1.2*U*_eq_(C).

### Crystal data

**Table:**

C_10_H_8_N_2_O	*F*(000) = 360
*M**_r_* = 172.18	*D*_x_ = 1.335 Mg m^−^^3^
Monoclinic, *P*2_1_/*c*	Mo *K*α radiation, λ = 0.71073 Å
Hall symbol: -P 2ybc	Cell parameters from 739 reflections
*a* = 5.704 (1) Å	θ = 3.3–26.1°
*b* = 8.557 (2) Å	µ = 0.09 mm^−^^1^
*c* = 17.595 (4) Å	*T* = 100 K
β = 94.382 (3)°	Irregular block, colorless
*V* = 856.4 (3) Å^3^	0.20 × 0.15 × 0.10 mm
*Z* = 4	

### Data collection

**Table:**

Bruker SMART APEX diffractometer	1207 reflections with *I* > 2σ(*I*)
Radiation source: fine-focus sealed tube	*R*_int_ = 0.042
graphite	θ_max_ = 27.5°, θ_min_ = 2.3°
ω scans	*h* = −6→7
4641 measured reflections	*k* = −11→10
1950 independent reflections	*l* = −22→21

### Refinement

**Table:**

Refinement on *F*^2^	Primary atom site location: structure-invariant direct methods
Least-squares matrix: full	Secondary atom site location: difference Fourier map
*R*[*F*^2^ > 2σ(*F*^2^)] = 0.046	Hydrogen site location: inferred from neighbouring sites
*wR*(*F*^2^) = 0.119	H-atom parameters constrained
*S* = 0.97	*w* = 1/[σ^2^(*F*_o_^2^) + (0.0572*P*)^2^] where *P* = (*F*_o_^2^ + 2*F*_c_^2^)/3
1950 reflections	(Δ/σ)_max_ = 0.001
118 parameters	Δρ_max_ = 0.20 e Å^−^^3^
0 restraints	Δρ_min_ = −0.27 e Å^−^^3^

### Fractional atomic coordinates and isotropic or equivalent isotropic displacement parameters (Å^2^)

**Table:**

	*x*	*y*	*z*	*U*_iso_*/*U*_eq_	
O1	0.0900 (2)	0.70888 (14)	0.45704 (7)	0.0301 (3)	
N1	0.4770 (3)	0.56153 (17)	0.60955 (8)	0.0281 (4)	
N2	0.4352 (3)	0.81790 (16)	0.51088 (8)	0.0252 (4)	
C1	0.0879 (3)	0.8138 (2)	0.39557 (9)	0.0244 (4)	
C2	−0.1015 (3)	0.9128 (2)	0.38509 (10)	0.0288 (4)	
H2	−0.2208	0.9130	0.4199	0.035*	
C3	−0.1150 (3)	1.0127 (2)	0.32238 (10)	0.0320 (5)	
H3	−0.2447	1.0819	0.3140	0.038*	
C4	0.0604 (3)	1.0112 (2)	0.27248 (10)	0.0309 (5)	
H4	0.0520	1.0803	0.2301	0.037*	
C5	0.2481 (3)	0.9097 (2)	0.28386 (10)	0.0298 (5)	
H5	0.3672	0.9086	0.2489	0.036*	
C6	0.2641 (3)	0.8093 (2)	0.34580 (9)	0.0266 (4)	
H6	0.3927	0.7393	0.3539	0.032*	
C7	0.2816 (3)	0.70451 (19)	0.50838 (9)	0.0225 (4)	
C8	0.2987 (3)	0.5762 (2)	0.55764 (10)	0.0279 (4)	
H8	0.1799	0.4981	0.5537	0.033*	
C9	0.6367 (3)	0.6767 (2)	0.61232 (10)	0.0275 (4)	
H9	0.7686	0.6707	0.6486	0.033*	
C10	0.6149 (3)	0.8027 (2)	0.56430 (9)	0.0274 (4)	
H10	0.7311	0.8823	0.5690	0.033*	

### Atomic displacement parameters (Å^2^)

**Table:**

	*U*^11^	*U*^22^	*U*^33^	*U*^12^	*U*^13^	*U*^23^
O1	0.0285 (7)	0.0309 (8)	0.0299 (7)	−0.0092 (6)	−0.0033 (5)	0.0105 (6)
N1	0.0356 (9)	0.0253 (8)	0.0237 (8)	−0.0023 (7)	0.0037 (7)	0.0021 (7)
N2	0.0304 (9)	0.0229 (8)	0.0222 (8)	−0.0055 (6)	0.0020 (6)	0.0000 (7)
C1	0.0279 (10)	0.0230 (10)	0.0215 (9)	−0.0088 (8)	−0.0028 (7)	0.0039 (8)
C2	0.0233 (10)	0.0323 (11)	0.0307 (10)	−0.0049 (8)	0.0021 (7)	0.0031 (9)
C3	0.0267 (11)	0.0321 (11)	0.0362 (10)	−0.0007 (8)	−0.0041 (8)	0.0044 (9)
C4	0.0338 (11)	0.0344 (11)	0.0237 (9)	−0.0062 (9)	−0.0037 (8)	0.0068 (9)
C5	0.0316 (11)	0.0368 (11)	0.0209 (9)	−0.0056 (9)	0.0011 (8)	−0.0027 (9)
C6	0.0280 (10)	0.0256 (10)	0.0255 (9)	−0.0014 (8)	−0.0020 (8)	−0.0032 (8)
C7	0.0253 (10)	0.0224 (10)	0.0203 (8)	−0.0029 (7)	0.0043 (7)	−0.0007 (8)
C8	0.0319 (11)	0.0246 (10)	0.0274 (10)	−0.0060 (8)	0.0048 (8)	0.0015 (8)
C9	0.0315 (11)	0.0287 (10)	0.0219 (9)	−0.0001 (8)	−0.0011 (7)	0.0005 (8)
C10	0.0295 (10)	0.0287 (11)	0.0238 (9)	−0.0076 (8)	0.0003 (7)	−0.0011 (9)

### Geometric parameters (Å, °)

**Table:**

O1—C7	1.364 (2)	C3—H3	0.9500
O1—C1	1.4050 (19)	C4—C5	1.382 (3)
N1—C8	1.320 (2)	C4—H4	0.9500
N1—C9	1.340 (2)	C5—C6	1.385 (2)
N2—C7	1.306 (2)	C5—H5	0.9500
N2—C10	1.343 (2)	C6—H6	0.9500
C1—C2	1.374 (3)	C7—C8	1.398 (2)
C1—C6	1.383 (2)	C8—H8	0.9500
C2—C3	1.394 (2)	C9—C10	1.370 (2)
C2—H2	0.9500	C9—H9	0.9500
C3—C4	1.381 (3)	C10—H10	0.9500
			
C7—O1—C1	119.11 (13)	C6—C5—H5	119.8
C8—N1—C9	116.18 (15)	C1—C6—C5	118.29 (17)
C7—N2—C10	115.21 (15)	C1—C6—H6	120.9
C2—C1—C6	122.28 (16)	C5—C6—H6	120.9
C2—C1—O1	117.23 (15)	N2—C7—O1	120.23 (15)
C6—C1—O1	120.38 (16)	N2—C7—C8	123.24 (16)
C1—C2—C3	118.68 (17)	O1—C7—C8	116.52 (15)
C1—C2—H2	120.7	N1—C8—C7	121.13 (16)
C3—C2—H2	120.7	N1—C8—H8	119.4
C4—C3—C2	119.90 (18)	C7—C8—H8	119.4
C4—C3—H3	120.0	N1—C9—C10	121.80 (16)
C2—C3—H3	120.0	N1—C9—H9	119.1
C3—C4—C5	120.36 (17)	C10—C9—H9	119.1
C3—C4—H4	119.8	N2—C10—C9	122.43 (17)
C5—C4—H4	119.8	N2—C10—H10	118.8
C4—C5—C6	120.49 (17)	C9—C10—H10	118.8
C4—C5—H5	119.8		
			
C7—O1—C1—C2	−126.78 (17)	C10—N2—C7—O1	179.05 (14)
C7—O1—C1—C6	56.9 (2)	C10—N2—C7—C8	0.5 (2)
C6—C1—C2—C3	−0.5 (3)	C1—O1—C7—N2	15.5 (2)
O1—C1—C2—C3	−176.75 (15)	C1—O1—C7—C8	−165.92 (14)
C1—C2—C3—C4	−0.2 (3)	C9—N1—C8—C7	0.6 (2)
C2—C3—C4—C5	0.8 (3)	N2—C7—C8—N1	−1.1 (3)
C3—C4—C5—C6	−0.7 (3)	O1—C7—C8—N1	−179.72 (15)
C2—C1—C6—C5	0.6 (3)	C8—N1—C9—C10	0.4 (3)
O1—C1—C6—C5	176.75 (14)	C7—N2—C10—C9	0.5 (2)
C4—C5—C6—C1	0.0 (3)	N1—C9—C10—N2	−1.0 (3)
